# Prenatal and postnatal maternal anxiety and amygdala structure and function in young children

**DOI:** 10.1038/s41598-021-83249-2

**Published:** 2021-02-17

**Authors:** Claire Donnici, Xiangyu Long, Deborah Dewey, Nicole Letourneau, Bennett Landman, Yuankai Huo, Catherine Lebel

**Affiliations:** 1 Neuroscience Program, University of Calgary, Calgary, AB Canada; 2 Alberta Children’s Hospital Research Institute, Calgary, AB Canada; 3 Department of Radiology, University of Calgary, Calgary, AB Canada; 4 Department of Pediatrics, University of Calgary, Calgary, AB Canada; 5 Hotchkiss Brain Institute, Calgary, AB Canada; 6 Department of Community Health Sciences, University of Calgary, Calgary, AB Canada; 7 Faculty of Nursing, University of Calgary, Calgary, AB Canada; 8 Department of Electrical Engineering & Computer Science, Vanderbilt University, Nashville, TN USA

**Keywords:** Neuroscience, Paediatric research

## Abstract

Anxiety symptoms are relatively common during pregnancy and are associated with behavioural problems in children. The amygdala is involved in emotion regulation, and its volume and function are associated with exposure to prenatal maternal depression. The associations between perinatal maternal anxiety and children’s amygdala structure and function remain unclear. The objective of this study was to determine associations between prenatal and postnatal maternal anxiety and amygdala structure and function in children. Maternal anxiety was measured during the second trimester of pregnancy and 12 weeks postpartum. T1-weighted anatomical data and functional magnetic resonance imaging data were collected from 54 children (25 females), between the ages of 3–7 years. Amygdala volume was calculated and functional connectivity maps were created between the amygdalae and the rest of the brain. Spearman correlations were used to test associations between amygdala volume/functional connectivity and maternal anxiety symptoms, controlling for maternal depression symptoms. Second trimester maternal anxiety symptoms were negatively associated with functional connectivity between the left amygdala and clusters in bilateral parietal regions; higher maternal anxiety was associated with increased negative connectivity. Postnatal maternal anxiety symptoms were positively associated with child amygdala volume, but this finding did not remain significant while controlling for total brain volume. These functional connectivity differences may underlie behavioral outcomes in children exposed to maternal anxiety during pregnancy.

## Introduction

Anxiety is relatively common during the perinatal period, with approximately 16% of women experiencing clinical levels of symptoms, compared to 7% of the general population^1,2^. This is particularly concerning because children born to mothers who experience high levels of anxiety during and after pregnancy are more likely to demonstrate increased internalizing behaviours such as anxiety symptoms and externalizing behaviours such as attention problems and aggression^3–8^.

The neurological underpinnings of these outcomes are not well understood; however, the amygdala has been frequently implicated^9^. The amygdala is a subcortical structure of the limbic system that plays a key role in bottom-up and top-down processes of emotional regulation as well as regulation of learning, memory, fear, and aggression^10,11^. It has been implicated in detecting and signaling information about motivationally salient stimuli and contributing to core affect^12^. Using magnetic resonance imaging (MRI), structural and functional differences in the amygdala have been linked to depression, anxiety, emotional problems and several neuropsychiatric disorders^10^.

Research investigating associations between perinatal mental health and amygdala structure in children has typically focused on prenatal maternal depressive symptoms or prenatal stress. Greater prenatal maternal depressive symptoms and increased cortisol are related to larger right amygdala volumes in girls at 4 and 7 years of age^9,13^. Pregnancy-related anxiety in mothers during the second trimester was correlated with larger left amygdala volume in girls at 4 years^14^. Preterm neonates of women with physician-diagnosed prenatal anxiety and/or depression showed reduced functional connectivity between the left amygdala and the thalamus, hypothalamus, fusiform and brainstem^15^. Second trimester depressive symptoms have also been associated with decreased right amygdala functional connectivity with the left orbitofrontal cortex and temporal pole, and decreased left amygdala connectivity with the anterior cingulate cortex, caudate, insula and putamen in 4-year-old girls^16^. Maternal cortisol during pregnancy, an indicator of prenatal stress, has been related to neonatal amygdala functional connectivity with regions in the temporal lobe, dorsolateral prefrontal cortex, precuneus and fusiform gyrus^17^. However, the opposite effects were found in girls and boys: in girls, stronger amygdala connectivity was associated with higher maternal cortisol, whereas in boys, weakened amygdala connectivity was associated with higher maternal cortisol^17^.

Anxiety and depression are often comorbid, but they are different disorders with distinct symptoms and outcomes^18^. Maternal anxiety and depression have distinct trajectories across the perinatal period and are associated with different risks for birth outcomes and behavioural problems^19–21^. Few studies have investigated the effects of prenatal anxiety, independent of depression, on children’s brain development, but prenatal maternal anxiety appears to affect child brain structure differently than prenatal depression. In one study, higher prenatal maternal anxiety was associated with lower fractional anisotropy (FA) in newborns in several brain areas, none of which were related to prenatal maternal depression^22^. Information on the unique effects of maternal anxiety on brain structure and function is limited. An understanding of these unique effects is important for informing the nature of anxiety-specific screening and interventions, if necessary, for mothers during pregnancy and for children in early life.

Maternal anxiety likely affects brain structure differently during the prenatal period compared to the postnatal period. Prenatal maternal anxiety has been hypothesized to play a role in the development of limbic brain regions through biological mechanisms including exposure to increased maternal cortisol, epigenetic regulation of enzymes responsible for the protective environment of the placenta, and altered expression of hypothalamic–pituitary–adrenal (HPA) axis-related genes in the amygdala^9,23,24^. Postnatal maternal anxiety is more likely to act through psycho-social pathways such as decreased maternal sensitivity, which can affect regulation of infant emotions^25,26^. Previous work has suggested different time periods of exposure to heightened maternal depression are related to different outcomes. Greater prenatal depression was associated with larger right amygdala volume in girls, whereas greater postnatal symptoms were associated with higher right amygdala FA in girls and the overall sample^13^. Determining how timing of exposure to maternal anxiety plays a role in brain development during childhood could better inform guidelines for anxiety screening and intervention in pregnancy and postnatally if intervention is needed.

Previous studies relating maternal mental health during the second trimester (14–26 weeks gestation) and postpartum to child brain structure and function^14,16,27,28^ provide a rationale for examining anxiety symptoms at these time points. Early childhood is a time of extensive brain development^29^ and a time when the behaviour problems associated with prenatal maternal anxiety often emerge^8,29^, which suggests looking at children’s brains during this time could provide important insight into how maternal anxiety could impact child brain development. Therefore, the objective of this study was to determine associations between prenatal and postnatal maternal anxiety and amygdala volume and functional connectivity in young children and examine if associations interacted with child sex. We studied 54 mothers who reported their anxiety during the 2nd trimester of pregnancy and 12 weeks’ postpartum, and their 54 children who underwent passive viewing functional MRI scans between the ages of 2–7 years. Given the results of previous work, we hypothesized that prenatal and postnatal anxiety would be associated with larger amygdala volumes and reduced functional connectivity between the amygdala and frontal regions.

## Results

At the time of their MRI scan, children (29 male/25 female) were 2.99–7.25 years of age (mean = 4.77, SD = 1.13 years, median = 4.76 years). Mothers ranged in age from 22–42 years at child birth (mean = 31.6, SD = 3.0, median = 31 years). Median maternal anxiety scores were 0.10 (range = 0–1.7; IQR = 0–0.3) in the second trimester and 0.10 (range = 0–2.4; IQR = 0–0.2) at 12 weeks postpartum. Edinburgh Postnatal Depression Scores (EPDS) scores were collected at the same time as anxiety symptoms; mean scores were 4.81 (SD = 3.99) in the second trimester and 4.64 (SD = 3.85) at 12 weeks postpartum. Participant characteristics are reported in Table 1.

**Table 1:** Descriptive information of participants.

	Range	Mean ± SD	Median [IQR]	*N*
**Children**				
Age at scan (years)	2.99–7.25	4.77 ± 1.13	4.76 [3.85–5.69]	54
Sex	25 female; 29 male			54
Gestational age at birth (weeks)	37–41.86	39.88 ± 1.11	39.93 [39.04–40.71]	54
Birth weight (g)	2850–4610	3442 ± 417	3429 [3101–3740]	54
**Mothers**				
Age at child’s birth (years)	22–42	31.6 ± 3.0	31 [30–33]	54
EPDS (second trimester)	0–16	4.81 ± 3.99	3 [2–6.75]	54
EPDS (12 weeks postpartum)	0–19	4.64 ± 3.85	4 [1.75–6.25]	50
SCL-90-R (second trimester)	0–1.7	0.24 ± 0.38	0.10 [0–0.3]	54
SCL-90-R (12 weeks postpartum)	0–2.4	0.16 ± 0.38	0.10 [0–0.2]	50

	n	(%)		
**Marital status**				
Cohabitating	5	9.26		
Married	47	87.0		
Single	2	3.70		
**Ethnicity**				
Caucasian	50	92.6		
Non-Caucasian	4	7.41		
**Education**				
Completed post-grad	20	37.0		
Completed university	23	42.6		
Completed trade, technical	11	20.4		
High school diploma	0	0		
Less than high school diploma	0	0		
**Household income**				
$100,000 or more	37	68.5		
$70,000–$99,999	12	22.2		
$40,000–$69,999	3	5.56		
$20,000–$39,999	2	3.70		
Less than $20,000	0	0		

EPDS, Edinburgh Postnatal Depression Scale; SCL-90-R, Symptom Checklist-90-Revised.

### Volume analysis

The volume analysis was performed with and without controlling for total brain volume. Second trimester maternal anxiety symptoms were not significantly associated with right or left amygdala volume. Maternal anxiety symptoms at 12 weeks postpartum were positively associated with right amygdala volume in children (n = 49; rho = 0.42; p = 0.0067, q = 0.027), without controlling for total brain volume. Postnatal maternal anxiety symptoms were also associated with left amygdala volumes prior to controlling for total brain volume, but this finding did not survive FDR correction (n = 49; rho = 0.33; p = 0.037, q = 0.073). After adding total brain volume as a covariate, postnatal maternal anxiety symptoms were associated with right amygdala volume, but this finding did not survive FDR correction (n = 49; rho = 0.35; p = 0.026, q = 0.10). Child left amygdala volume was not associated with postnatal maternal anxiety after controlling for total brain volume (rho = 0.22; p = 0.18).

### Functional connectivity analysis

**Figure 1:**
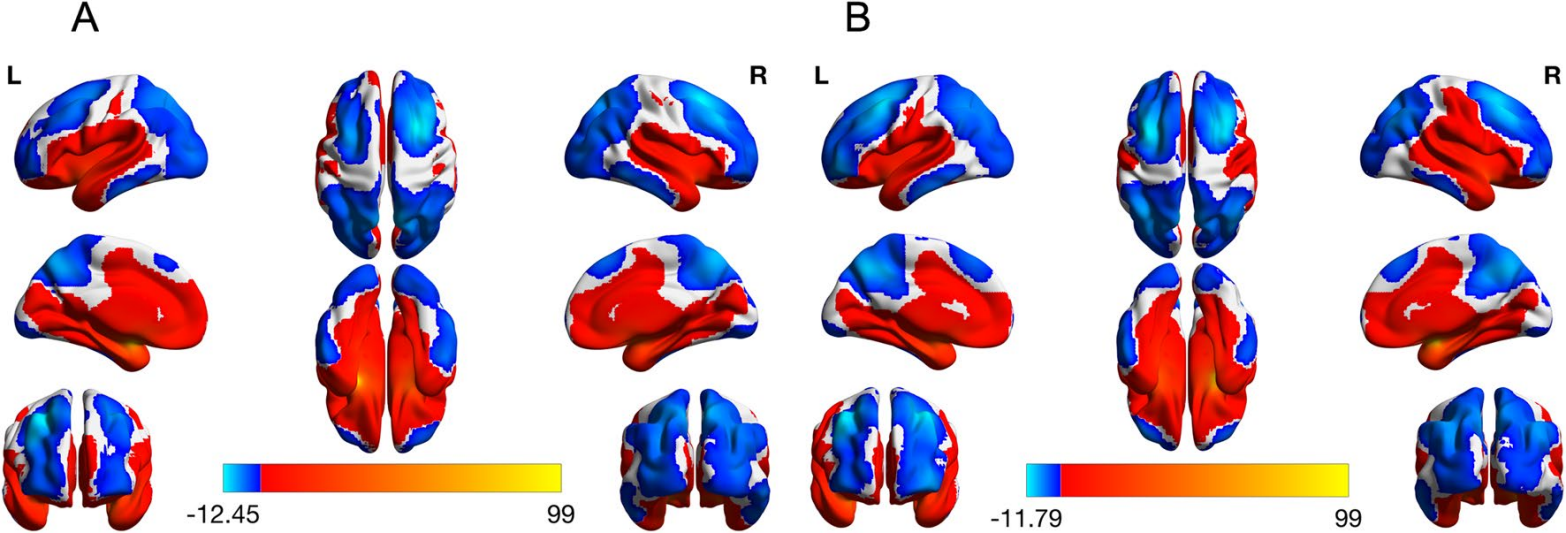
Left and right amygdala functional connectivity in the whole sample. Left and right amygdala functional connectivity with the rest of the brain was determined using a one sample t-test (p < 0.05, corrected). Both the left and right amygdala showed positive connectivity with the medial frontal cortex, middle and anterior cingulate as well as the superior temporal cortex and sensorimotor areas. Positive connectivity to the basal ganglia, the hippocampus, the fusiform and the insula was also observed. Areas with negative correlation to the left and right amygdala included the lateral frontal cortex, parietal lobe and occipital cortex in addition to the precuneus, posterior cingulate and a region of the inferior temporal cortex (left amygdala connectivity is pictured in (**A**), while right amygdala connectivity is pictured in (**B**)).

Amygdala functional networks are shown in Fig. 1. Both the left and right amygdala have positive connectivity to nearby regions including the basal ganglia, the hippocampus and the fusiform. Positive connectivity extended further to the insula, the superior temporal cortex, the sensorimotor cortex, and through the middle/anterior cingulate into the medial frontal cortex. A small portion of the parietal lobe also had positive connectivity with the amygdala. Areas with negative functional connectivity to the amygdala included the lateral frontal cortex, the parietal lobe, and the occipital cortex. Negative functional connectivity to the amygdala was also observed in the inferior temporal cortex, the precuneus, and the posterior cingulate. These observations are consistent with previous work investigating resting state functional connectivity of the amygdala in children/youth aged 4–23 years and in adults^30,31^.

**Figure 2:**
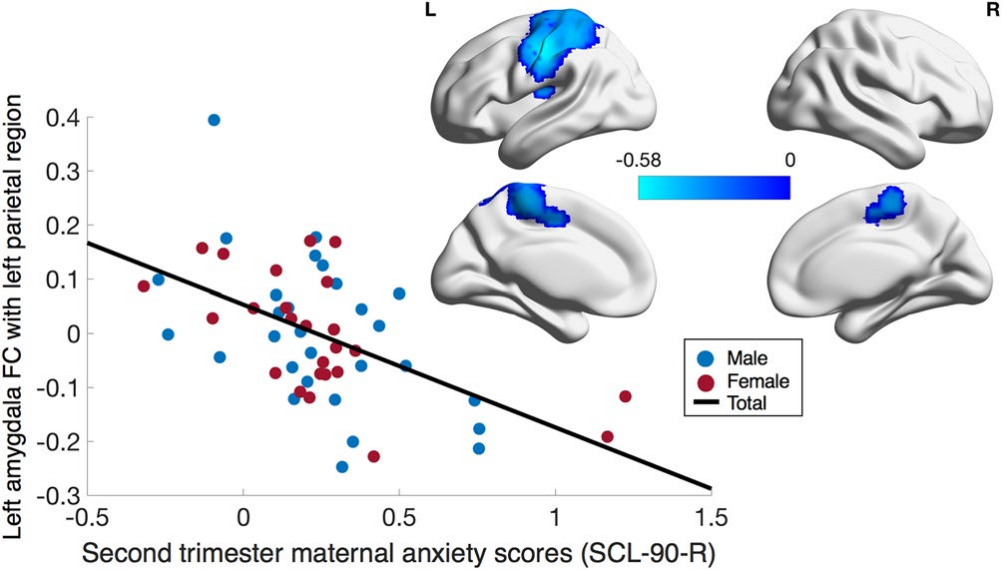
Relationship between maternal prenatal anxiety and amygdala functional connectivity. Second trimester SCL-90-R scores were negatively correlated with functional connectivity between the left amygdala and one large cluster encompassing several brain regions shown in blue (rho = − 0.50, p = 1.2 × 10^–4^). This effect was seen when controlling for child’s age at the scan, sex, birth weight, gestational age at birth, household income, and maternal prenatal depressive symptoms. The data plotted are residuals of anxiety scores and cluster-averaged left amygdala functional connectivity after accounting for covariates. Results were corrected for multiple comparisons at voxelwise and cluster p < 0.05 (cluster size: 2857 voxels; Peak: − 49, − 23, 36—left inferior parietal lobule) (N = 54).

Using a voxel threshold of p < 0.05, maternal prenatal anxiety symptoms were negatively correlated with functional connectivity between the left amygdala and one large cluster centered in the left parietal region (2857 voxels; rho = − 0.50; p = 1.2 × 10^–4^), such that children of mothers with higher prenatal anxiety tended to have more negative connectivity between the amygdala and these areas. This finding was observed when controlling for age, sex, gestational age at birth, birth weight, household income and second trimester maternal depressive symptoms. The cluster encompassed parts of the left supplementary motor area, the postcentral and precentral gyri, superior and inferior parietal lobules, and the insula, thalamus and putamen (Fig. 2). The cluster extended medially into the left paracentral lobule and the right paracentral lobule, right supplementary motor area and right middle cingulate (Fig. 2), with a peak in the left inferior parietal lobule (rho =  − 0.56; p = 1.0 × 10^–5^). Local maxima were observed in the left insula, left paracentral lobule, left superior frontal gyrus, left supplementary motor area, and right middle cingulate (Table 2). No significant sex-anxiety or age-anxiety interactions were observed and no significant correlations between right amygdala functional connectivity and second trimester anxiety scores were found.

**Table 2:** Local maxima in clusters found through Spearman correlation of anxiety scores and amygdala functional connectivity.

Area	MNI coordinates at local maximum	Spearman’s rho at local maximum	p value
**Analysis 1**^**a**^			
Cluster 1			
Left inferior parietal lobule	− 49, − 23, 36^c^	− 0.56	1.0 × 10^–5^
Left insula	− 28, − 23, 12	− 0.38	3.7 × 10^–3^
Left paracentral lobule	− 4, − 32, 63	− 0.40	2.9 × 10^–3^
Left superior frontal gyrus	− 22, − 8, 45	− 0.40	2.6 × 10^–3^
Left SMA	− 13, − 5, 45	− 0.35	1.0 × 10^–2^
Right middle cingulate	14, − 11, 48	− 0.36	7.2 × 10^–3^
**Analysis 2**^**b**^			
Cluster 1			
Left postcentral gyrus	− 46, − 17, 39^c^	− 0.59	1.0 × 10^–5^
Left superior parietal lobule	− 37, − 41, 54	− 0.53	6.0 × 10^–5^
Left precentral gyrus	− 28, − 17, 54	− 0.48	4.6 × 10^–4^
Cluster 2			
Right postcentral gyrus	50, − 11, 30^c^	− 0.53	9.0 × 10^–5^
Right inferior parietal lobule	50, − 32, 54	− 0.50	2.3 × 10^–4^
Right superior frontal gyrus	20, − 5, 48	− 0.49	2.8 × 10^–4^

Cluster 1: 2857 voxels (rho = − 0.50; p = 1.2 × 10^–4^); Cluster 2: 2275 voxels (rho  = − 0.51; p = 1.8 × 10^–4^); Cluster 3: 2155 voxels (rho  = − 0.52; p = 1.1 × 10^–4^). These data are representative of voxel-level results.

^a^Analysis 1 included age, sex, gestational age at birth, birth weight, household income and second trimester maternal depression as covariates.

^b^Analysis 2 included age, sex, gestational age at birth, birth weight, household income, second trimester maternal depression as well as postnatal maternal depression and anxiety symptoms as covariates.

^c^Cluster peak.

**Figure 3:**
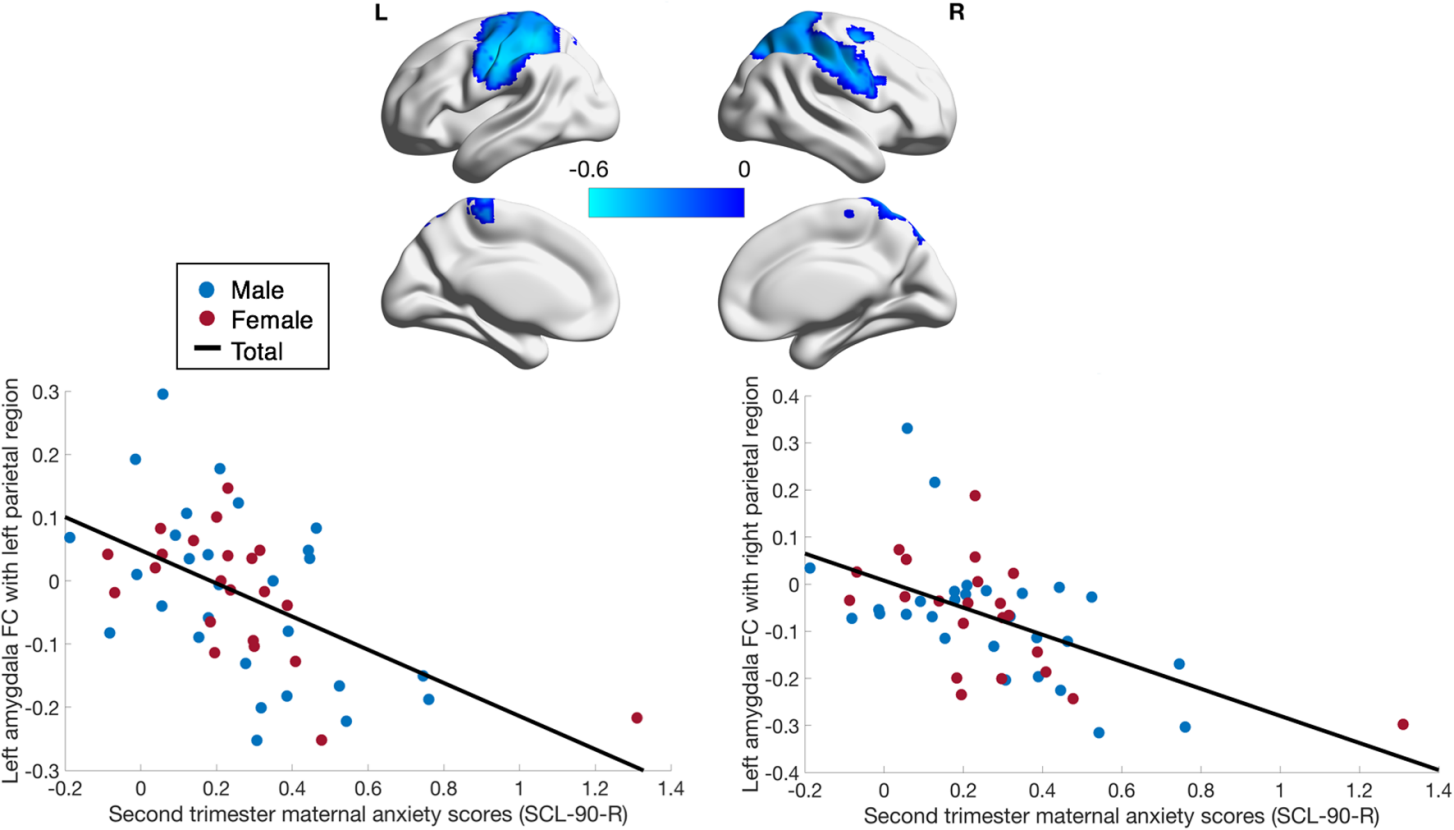
Relationship between maternal prenatal anxiety and amygdala functional connectivity controlling for postpartum depression and anxiety.  A correlation analysis between second trimester SCL-90-R scores and left amygdala functional connectivity controlling for postnatal anxiety (SCL-90-R) and depression (EPDS), in addition to child’s age, sex, birth weight, gestational age at birth, household income and prenatal depressive symptoms was performed. Second trimester SCL-90-R scores were negatively correlated with functional connectivity between the left amygdala and two clusters in the left and right parietal regions both shown in blue. The data plotted are residuals of anxiety scores and cluster-averaged left amygdala functional connectivity after accounting for covariates. Prenatal anxiety scores and connectivity between the left amygdala and the left parietal region is plotted on the left (Cluster size: 2275 voxels; rho = − 0.51, p = 1.8 × 10^–4^; Peak: − 46, − 17, 39—left postcentral gyrus) while connectivity between the left amygdala and right parietal region is plotted on the right (Cluster size: 2155 voxels; rho = − 0.52, p = 1.1 × 10^–4^; Peak: 50, − 11, 30—right postcentral gyrus). Results were corrected for multiple comparisons at voxelwise and cluster p < 0.05 (*n* = 50).

When controlling for postnatal depression and anxiety (as well as all original covariates), second trimester maternal anxiety was negatively associated with functional connectivity between the left amygdala and most of the same regions previously mentioned (n = 50), encompassing one large cluster (2275 voxels; rho =  − 0.51; p = 1.8 × 10^–4^) (Fig. 3). The cluster did not include the left insula, thalamus, or putamen. The peak of this cluster was located in the left postcentral gyrus (rho =  − 0.59; p = 1.0 × 10^–5^) and other local maxima were found in the left superior parietal lobule and left precentral gyrus (Table 2). In this analysis, second trimester maternal anxiety symptoms were also negatively correlated to left amygdala functional connectivity with a cluster in the right parietal region (Fig. 3). This cluster included the right postcentral gyrus, right precentral gyrus, right inferior parietal lobule and the right supramarginal gyrus. The cluster also reached the right middle and superior frontal gyri (2155 voxels; rho =  − 0.52; p = 1.1 × 10^–4^). The cluster peak was in the right postcentral gyrus (rho =  − 0.53; p = 9.0 × 10^–5^). Local maxima were found in the right inferior parietal lobule and right superior frontal gyrus (Table 2). Sex-anxiety and age-anxiety interactions remained non-significant.

Combined with the functional connectivity maps (Fig. 1), these results suggest that as anxiety symptoms increase, amygdala functional connectivity with the clusters shifts from no coupling or weak positive/negative coupling to increased negative coupling.

We repeated the above analyses with a voxel threshold of p < 0.001. Second trimester maternal anxiety was significantly associated with a similar but smaller cluster in the left inferior parietal lobe and the left postcentral gyrus (233 voxels; rho =  − 0.58, p = 4.0 × 10^–6^; Peak:  − 49,  − 23, 36). After controlling for postpartum maternal depression and anxiety, the cluster was slightly smaller, but still included the left postcentral gyrus and left inferior parietal lobe (190 voxels; rho =  − 0.56, p = 2.0 × 10^–5^; Peak:  − 46,  − 17, 39) (Supplementary Figure 1). The cluster previously found between the left amygdala and right parietal area did not remain significant after applying the more stringent uncorrected threshold.

There were no significant correlations between children’s left or right amygdala functional connectivity and postnatal maternal anxiety symptoms when controlling for age, sex, gestational age at birth, birth weight, household income, prenatal maternal anxiety and depressive symptoms, and postnatal maternal depressive symptoms.

## Discussion

Here we show that prenatal maternal anxiety is significantly related to functional connectivity of the left amygdala in young children. Relationships were independent of maternal depressive symptoms, and demonstrate differential associations for prenatal and postnatal anxiety. Disrupted amygdala functional connectivity may underlie child outcomes such as internalizing and externalizing behaviours like emotional problems and problems with attention. Therefore, recognizing and treating maternal anxiety may be important to children’s long-term behaviour and mental health.

Higher second trimester anxiety was associated with more negative connectivity between the left amygdala and clusters that had peaks in the left inferior parietal lobe and left and right postcentral gyri (Table 2). The primary function of the somatosensory cortex is the processing of sensory stimuli, but it is also involved in emotional processing, including generation of affective state and re-evaluation of stimuli when determining emotional salience^32^. A previous study in children aged 9–14 years showed that more negative left amygdala—postcentral gyrus connectivity was related to more externalizing symptoms in children^33^. Thus, the increased negative left amygdala—parietal connectivity seen here may be a potential mechanism that underlies the association between maternal anxiety/stress and externalizing behaviours that has been noted in literature^3^. Future imaging studies with larger samples that include behavioural measures are needed to investigate this.

When controlling for postpartum anxiety and depression, similar relationships were seen between prenatal maternal anxiety and children’s functional brain connectivity, though significant regions included more areas of the right parietal lobe. Shyness is negatively correlated with functional connectivity between the left amygdala and the right inferior parietal lobule^34^, and prenatal maternal anxiety has been related to shy/inhibited behaviours^35,36^. While childhood shyness has been associated with internalizing behaviours and social phobia later in development, it has also been shown to be protective against generalized anxiety disorder and depression in girls^36^. Decreased left amygdala—right parietal functional connectivity may underlie the relationship between maternal anxiety symptoms and childhood behaviours such as shyness. Decreased amygdala functional connectivity to the right superior frontal gyrus and superior parietal cortex has been found in patients with major depressive disorder and their relatives, suggesting another link between the amygdala functional connectivity relationships observed in our study and internalizing behaviours^37^. Reduced connectivity between the amygdala and parietal cortex has additionally been related to emotion recognition^38^. During early childhood, internalizing and externalizing behaviors have a high rate of comorbidity^39^ and are associated with similar brain areas^40^. Prenatal maternal stress has been related to internalizing and externalizing behaviors^3^. The altered amygdala functional connectivity seen in this work could be related to either or both types of behavior; however, further analyses that include behavioural scores would be required to confirm this.

Many of the other brain areas where connectivity with the amygdala was negatively associated with anxiety are involved in emotional regulation^41,42^. The insula has a role in visceral sensory and motor responses as well as emotional perception and subjective feelings^43^. In youth and adolescents aged 10–17 years, increasing severity of behavioural and emotional dysregulation has been linked to decreased functional connectivity between the amygdala and posterior insula^44^. Higher emotional dysregulation in patients with social and generalized anxiety disorders has also been associated with stronger negative functional connectivity between the left amygdala and areas of the cingulate cortex^41^.

Amygdala functional connectivity with subcortical and limbic regions is largely stable between 4 years of age and early adulthood^30^. However, amygdala functional connectivity with areas including the pre- and post-central gyri, precuneus, cingulate gyri, left inferior parietal lobe and left insula becomes more negative from childhood to adulthood^30^. Therefore, the stronger negative functional connectivity seen here in children born to mothers with higher anxiety scores may suggest early maturation of these functional connections.

Our findings are similar to those observed with prenatal maternal depression and prenatal cortisol, including altered amygdala functional connectivity with the insula, thalamus, putamen and cingulate cortex in children^15,16,45,46^. However, we found prenatal maternal anxiety was associated with amygdala-parietal connectivity, whereas previous studies relate prenatal maternal depression symptoms with functional connectivity between the amygdala and frontal regions^16,45,46^. Similar to our amygdala functional connectivity results, previous work has suggested that early life stress is associated with accelerated development of amygdala functional connectivity with areas including the prefrontal cortex^47,48^. Thus, our results suggest that prenatal anxiety may exert unique effects on fetal brain development, and that it is important to consider prenatal maternal anxiety symptoms separately from depression when examining effects on children’s brains.

We observed a significant relationship between maternal anxiety and left but not right amygdala functional connectivity. The left amygdala is suggested to be involved in affective information encoding related to language and emotional processing of fearful stimuli^49^. Further, Ochsner et al. demonstrated that top-down responses modulate left amygdala and not right amygdala activity, which they suggested may indicate that the left amygdala is more susceptible to the effect of top-down inputs in emotion regulation and anxiety^11^, though fMRI studies on the lateralization of emotion in the brain have shown mixed results^50^.

The mechanisms through which prenatal maternal mental health affect functional connectivity remain unclear, but could include epigenetic regulation and exposure to glucocorticoids. Significant positive correlations have been reported between prenatal maternal anxiety and maternal cortisol during each trimester of pregnancy^51^. The amygdala has glucocorticoid and mineralocorticoid receptors and therefore can be directly affected by cortisol release^52^. Glucocorticoid levels, including levels of cortisol, normally rise throughout pregnancy, and are important for typical brain development, but exposure to excess maternal glucocorticoids can dysregulate the infant stress response^53^. Multiple studies have shown that maternal distress and anxiety are also linked to epigenetic regulation^54,55^, which via DNA methylation and histone modification, can result in higher glucocorticoid exposure^54,55^. Epigenetic regulation of genes encoding cortisol-binding receptors and cortisol inactivating enzymes can be influenced by maternal anxiety and depression, and increased methylation of these genes can increase the salivary cortisol stress response in infants^53^.

We observed a positive association between postnatal maternal anxiety and child right amygdala volumes. The relationship remained significant after controlling for total brain volume but did not survive FDR correction. Previous studies have shown that higher prenatal maternal stress and depression are associated with larger amygdala volume in children aged 4.5–11 years^13,27^. Furthermore, higher levels of cortisol in early pregnancy, which typically reflect higher stress, have been associated with larger right amygdala volume in children aged 7 years^9^. Postnatal depressive symptoms have been significantly correlated to larger amygdala volumes^56^ and higher fractional anisotropy in children^13^. Larger amygdala volumes in children exposed to prenatal depression and stress have been associated with more affective or externalizing problems^9,27^. It is possible that the relationship between postnatal maternal anxiety and amygdala volume may be more pronounced in children born to mothers with higher anxiety symptoms, but less apparent in children of mothers with lower anxiety symptoms, such as the population studied here.

Postnatal maternal anxiety likely acts through a psycho-social pathway. Maternal anxiety can lead to decreased maternal-child relationship quality, including poor maternal sensitivity and responsiveness and insecure attachment, important regulators of the infant stress response during early life^26,57,58^. The amygdala’s rapid growth in the postnatal period^59^ likely makes it very sensitive to environmental exposures^60^ such as parenting, which may be reflected by the relationship between postnatal anxiety and amygdala volume observed here.

We found no significant sex differences or sex-anxiety interactions. Some previous studies have reported stronger effects of prenatal depression and cortisol levels on amygdala volume and functional connectivity in girls^9,13,16,27^, while others have not reported sex differences^45,56^. Sex differences may depend on the timing of stress exposure and/or the age of the children being examined. Increased first trimester maternal emotional complaints (summed measures of depression and anxiety) have been associated with more internalizing behaviours in boys, whereas more third trimester emotional complaints have been associated with internalizing and externalizing behaviours in girls^61^. Additionally, neurodevelopmental trajectories differ for boys and girls, potentially leaving male and female brains differentially vulnerable to prenatal maternal anxiety and depression^30,59^.

## Limitations

Functional data were collected while children were watching movies. While passive viewing fMRI data cannot be exactly equated to resting state fMRI, movies or videos greatly increase compliance and significantly reduce head motion in MRI scans of children aged 3–7 years^62^, and have been used in previous studies of young children^63–65^. Functional brain networks are generally similar during passive viewing compared to rest, but there are some differences that have been reported in visual networks and dorsal attention networks^62,66^. These differences are unlikely to have impacted the left and right amygdala networks studied here. In our study, most mothers had mild anxiety symptoms. This limits the range of scores, but also indicates that the relationship between maternal prenatal/postnatal anxiety and their children’s amygdala structure and function is present even in women without severe anxiety disorders. The sample of women in this study were from a low sociodemographic risk population and it may not be appropriate to generalize these findings to a sociodemographically diverse population. However, the relatively high measures of socioeconomic status and the limited ethnic diversity seen in our sample reveal that even in populations with fewer risk factors for maternal adversity, higher levels of anxiety might lead to altered brain development in children. The only prenatal anxiety symptoms included in this analysis were from the second trimester. While some women completed questionnaires in the first and/or third trimesters, the sample sizes were not sufficient to examine these relationships. Future studies looking at first trimester and third trimester symptoms would provide a more complete picture for mothers and health care providers of when intervention would be most efficacious. Furthermore, postpartum maternal anxiety scores only included a measurement at 12 weeks, which does not consider whether maternal anxiety persisted throughout childhood. While the purpose of the analyses presented here was to examine impacts of timing of exposure in the prenatal and early postnatal period, future studies could explore longitudinal trajectories of maternal anxiety and impacts on brain structure. Further, future studies including behaviour scores are necessary to provide clarity on how results may underlie behaviour.

## Conclusions

Our findings demonstrate a significant relationship between prenatal maternal anxiety and child left amygdala functional connectivity. We also found an association between postnatal maternal anxiety and child right amygdala volume, though this did not persist after controlling for total brain volume. Both findings appear to suggest greater developmental maturity in children exposed to higher maternal anxiety symptoms, which may not be optimal in this case. The larger amygdala volumes and more negative functional connectivity observed here are consistent with findings in children, adolescents, and adults with internalizing and externalizing behaviours, emotional regulation problems, anxiety and depression^9,27,33,37,38,44^, indicating that changes to the amygdala may be a mechanism via which perinatal anxiety increases vulnerability for behavioural and mental health problems later in a child’s life. Ultimately, these findings emphasize the importance of screening for and treating not only depression, but also anxiety in women during pregnancy to promote healthy outcomes for mothers and their children.

## Methods

### Study design and participants

This study reports data from 54 mother–child pairs. Women were recruited in pregnancy as part of the ongoing Alberta Pregnancy Outcomes and Nutrition (APrON) study^67^. From this cohort, we recruited 117 children who underwent neuroimaging in early childhood^68^; 63 were excluded for various reasons: missing maternal anxiety scores (n = 25), incomplete fMRI scans (n = 17), excessive motion during scans (n = 12), incidental findings (n = 5), sleeping during the scan (n = 3), or confirmed diagnosis with a developmental disorder after scanning took place (n = 1). The 54 children in the current study were free of diagnosed neurodevelopmental, neurological, or genetic disorders, as well as contraindications to magnetic resonance imaging (MRI). Informed consent was obtained from mothers at the time of prenatal anxiety data collection; informed parental consent was obtained at the time of child MRI acquisition. This study was approved by the University of Calgary Conjoint Health Research Ethics Board (CHREB) and is in accordance with the guidelines of the Declaration of Helsinki.

### Anxiety and depression measures

Maternal anxiety symptoms were measured using the 10-question anxiety component of the Symptom Checklist 90-Revised (SCL-90-R) questionnaire during the second trimester of pregnancy (17 ± 2 weeks) and at 12 weeks postpartum^67,69^. The SCL-90-R evaluates a broad range of psychological problems and symptoms of psychopathology^69^. The 10-question anxiety component was used for this study to obtain a generalized measure of anxiety and to reduce participant burden with a shorter questionnaire. This subscale demonstrates good convergent validity and internal consistency (Cronbach’s α = 0.81). Convergent validity of the SCL-90-R has been demonstrated by several studies where SCL-90-R symptom dimensions correlated with the expected constructs of the clinical interviews, personal history and additional psychiatric measures^70–72^. Items include “feeling distressed by nervousness or shakiness inside,” “suddenly scared for no reason,” “feeling fearful,” “trembling,” “feeling tensed or keyed up,” “spells of terror or panic,” “feeling so restless you couldn’t sit still,” “thoughts and images of frightening nature,” “heart pounding or racing,” and “feeling that something bad is going to happen.” Items were answered on a scale from 0 to 4 based on the degree to which a respondent experienced a given symptom (0: not at all, 1: a little bit, 2: moderately, 3: quite a bit, and 4: extremely). Responses were totaled and averaged to provide a composite score that ranged from 0 to 4; higher scores are associated with higher anxiety symptoms^67,69^. Averages were calculated for participants who answered at least 8 of 10 questions.

Depressive symptoms were measured at the same time as anxiety symptoms during the second trimester and at 12 weeks postpartum using the Edinburgh Postnatal Depression Scale (EPDS), a 10-item self-administered questionnaire validated for assessment of prenatal and postnatal depressive symptoms^73^. Maternal depressive symptoms were included as a covariate because prenatal and postnatal maternal depression have been associated with amygdala volume and functional connectivity in children^13,16,45,46^.

### Image acquisition

MRI data were collected using a GE 3T MR750w (General Electric, Waukesha, WI) scanner with a 32-channel head coil at the Alberta Children’s Hospital in Calgary, Alberta. Passive viewing fMRI data were collected using a gradient-echo echo-planar imaging (EPI) sequence; TR = 2 s, TE = 30 ms, flip angle = 60°, 36 slices, resolution = 3.59 × 3.59 × 3.6 mm, matrix size = 64 × 64, 250 volumes. These data were conceptualized as resting state fMRI data. T1-weighted images were obtained with an FSPGR BRAVO sequence; flip angle = 12°, 210 slices, TR = 8.23 ms, TE = 3.76 ms, resolution = 0.9 × 0.9x0.9 mm^3^, matrix size = 512 × 512, inversion time = 540 ms. Children were awake and watching self-selected movies for the duration of the scan^74^. Children were monitored to ensure they were awake throughout the scan.

### Amygdala segmentation and volume extraction

**Figure 4:**
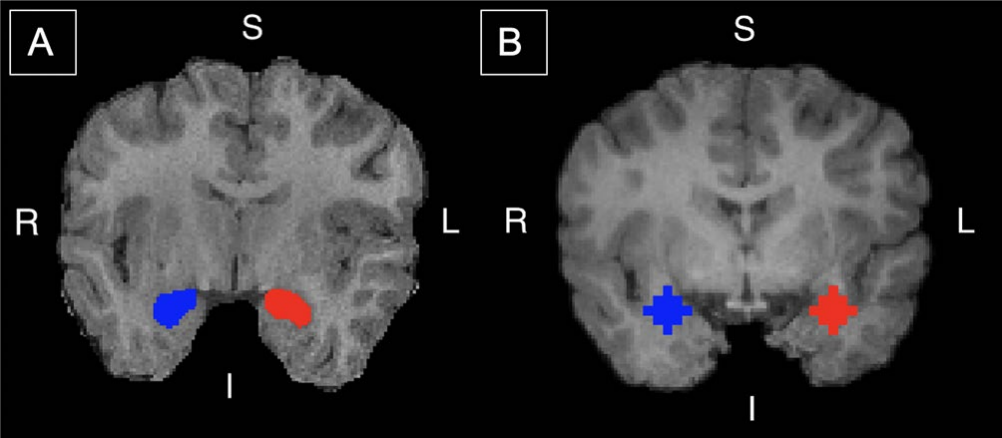
Amygdala segmentation and regions of interest. (**A**) Segmentations of the right (blue) and left (red) amygdala. Segmentations of each participant’s brain were obtained from raw T1 images with Multi-atlas Cortical Reconstruction Using Implicit Surface Evolution (MaCRUISE) software and were used to analyze the relationship between maternal anxiety and child amygdala volume. (**B**) Amygdala seed regions used in the functional connectivity analysis. Regions are overlayed on a T1 image registered to a pediatric brain template in standard space. Seed regions were defined with a 6 mm radius sphere centered at − 25, 1, − 18 (red; left amygdala) and 26, 1, − 18 (blue; right amygdala). Coordinates were obtained from the Automated Anatomical Labeling atlas (AAL). S—superior; I—inferior; R—right; L—left. The segmentations and regions of interest are displayed on the same participant.

Brain image segmentations were completed using Multi-atlas Cortical Reconstruction Using Implicit Surface Evolution (MaCRUISE) software at the Vanderbilt University Institute of Imaging Science, Center for Computational Imaging^75^. MaCRUISE software obtains self-consistent brain segmentations of 132 regions, including the right and left amygdala (Fig. 4), and cortical surfaces from T1-weighted MR images without compromising surface accuracy. Segmentations were manually quality-checked and edited using ITK_SNAP 3.8.0 where necessary^76^. Volumes of the right and left amygdala were extracted using the volumetric labels from the multi-atlas segmentation in MATLAB.

### Functional data preprocessing

T1 images were skull-stripped and segmented by tissue-type (white matter, grey matter, and cerebrospinal fluid (CSF)) to create individual masks. T1 images were registered to a pediatric brain template (ages 37–47 months) in Montreal Neurological Institute (MNI) standard space in order to align individual participant images^77^. T1 image preprocessing was completed in FSL^78^.

The first 10 volumes of fMRI data were removed to allow for signal stabilization. Data were corrected for slice timing and head motion to account for spatial misalignment between volumes. Data were then co-registered to that participant’s T1-image and underwent linear de-trending. The relative root-mean-square frame-wise displacement (FD) and its mean were calculated.

High relative FD was used to identify spike volumes and a matrix of these volumes was created. Datasets with FD higher than 0.25 mm or spike volumes that made the signals shorter than 5 min were excluded. The threshold of 0.25 mm is an empirical value chosen based on previous studies^79,80^. Preprocessing of fMRI images included head motion, white matter signal, cerebrospinal fluid signal and global signal regression. A model with 36 parameters was formed using the average signals from whole brain, CSF and white matter masks, 6 head motion parameters, their temporal derivatives and quadratic term signals^80^. These parameters and the spike matrix were regressed out of the pre-processed fMRI signals. fMRI signals were then band-pass filtered to remove unwanted signal components at low and high frequencies (0.009–0.08 Hz) and were transformed to MNI space using the pediatric brain template as well as resampled to 3 × 3 × 3 mm^3^. fMRI processing was completed using AFNI_18.1.12^81^. Data were spatially smoothed with a 6 mm full width at half maximum (FWHM) kernel in FSL.

### Functional connectivity maps

A 6 mm radius sphere was used to define the right and left amygdala as regions of interest, based on the Automated Anatomical Labeling (AAL) atlas and the location of the center of the amygdala^82^ (Right amygdala: 26, 1,  − 18; Left amygdala:  − 25, 1,  − 18, MNI space) (Fig. 4). A 6-mm radius sphere was chosen to capture the location of the amygdala in previous work investigating amygdala functional connectivity, which helped inform this analysis^83,84^. Average BOLD time series were extracted from the amygdala seed region in each participant and individual seed-based functional connectivity maps to the rest of the brain were created and transformed to Z-scores using Fisher’s r-to-Z transformation. Z-transformed functional connectivity maps were combined to create two 4D functional connectivity maps representing left and right amygdala functional networks for all participants.

### Statistical analysis

Statistical analysis was conducted in AFNI and MATLAB 2018A. In the first analysis, the relationships between maternal anxiety (second trimester and 12 weeks postpartum) and right and left amygdala volume were analyzed using Spearman’s partial correlations, controlling for maternal depressive symptoms, child’s gestational age at birth, birth weight, household income, age, and sex. Gestational age at birth and birth weight have been linked to maternal prenatal anxiety and stress as well as structural development of child brain regions associated with the stress response^85–87^. Household income is a component of socioeconomic status, which can impact brain development^88^. In a second analysis, we controlled for total child brain volume in addition to age and all original covariates. Sex-anxiety and age-anxiety interaction terms were also included in the models, but were removed where no significant effects were present. The Benjamini and Hochberg false discovery rate (FDR) was used to correct for four multiple comparisons (2 anxiety timepoints for right and left amygdala volumes) at q < 0.05^89^.

A one sample t-test was used to determine functional connectivity between the left and right amygdala and the rest of the brain with a voxelwise p value of 0.05. Spearman’s correlations were performed to test the association of this connectivity with prenatal and postnatal maternal anxiety symptoms, as depression and anxiety scores were non-normally distributed according to the Kolmogorov–Smirnov test. 4D maps were correlated with second trimester anxiety scores, covarying for child’s age at scan, sex, birth weight, gestational age at birth, household income and maternal second trimester EPDS scores. In a subsequent analysis, maternal postnatal (12 weeks) EPDS and postnatal anxiety (SCL-90-R) scores were added as additional covariates (n = 50). A similar analysis was used to test the association between children’s right and left amygdala functional connectivity with the rest of the brain and maternal postnatal anxiety, controlling for child age, sex, gestational age at birth, birth weight, household income, maternal prenatal anxiety and prenatal and postnatal depressive symptoms. To correct for multiple comparisons, the AFNI command 3dFWHMx with the –acf option was used to obtain smoothness parameters and estimate cluster size at alpha < 0.05 via Monte Carlo simulation by 3dClustsim^81^. In the first analysis (N = 54), correlation maps were thresholded at rho > 0.2876 (voxelwise p-value of 0.05) with a cluster threshold of 2058 voxels. In the analysis controlling for maternal postnatal depression and anxiety, correlation maps were thresholded at rho > 0.3081 (voxelwise p-value of 0.05) with a cluster threshold of 2085 voxels. Significant clusters and amygdala functional connectivity to cluster regions were extracted for each participant. To examine local maxima, functional connectivity values and rho constants were extracted directly from individual coordinates. Results were displayed using BrainNet Viewer^90^. An uncorrected threshold of p < 0.05 was initially chosen based on other studies and to reduce Type II errors^15,63^. These analyses were also performed with a higher uncorrected threshold of p < 0.001 (rho > 0.4647, cluster threshold = 172 voxels; rho > 0.4950, cluster threshold = 165 voxels for analysis correcting for postpartum depression and anxiety symptoms).

## Supplementary Information

Supplementary Information.
