# Supplementary material for: Prenatal and postnatal maternal anxiety and amygdala structure and function in young children
Source: Sci Rep. 2021 Feb 17;11:4019. doi: 10.1038/s41598-021-83249-2 (PMC7889894; doi:10.1038/s41598-021-83249-2)
Supplement: Supplementary file 1 — Supplementary Information. [file 41598_2021_83249_MOESM1_ESM.docx]

**Prenatal and postnatal maternal anxiety and amygdala structure and function in young children**

Claire Donnici BHSc^1^, Xiangyu Long PhD^2,3^, Deborah Dewey PhD^2,4,5,6^, Nicole Letourneau PhD^1,2,4,6,7^, Bennett Landman PhD^8^, Yuankai Huo PhD^8^, Catherine Lebel PhD*^2,3,5^

**Affiliations:**

^1^Cumming School of Medicine, University of Calgary, Calgary AB

^2^Alberta Children’s Hospital Research Institute, Calgary AB

^3^University of Calgary Department of Radiology, Calgary AB

^4^University of Calgary Department of Pediatrics, Calgary AB

^5^Hotchkiss Brain Institute, Calgary AB

^6^University of Calgary Department of Community Health Sciences, Calgary AB

^7^University of Calgary Faculty of Nursing, Calgary AB

^8^Vanderbilt University Department of Electrical Engineering & Computer Science, Nashville TN

***Corresponding author:** Catherine Lebel, Ph.D., University of Calgary, Alberta Children’s Hospital, Room B4-513, 28 Oki Drive NW, Calgary, Alberta T3B6A8, Canada; E-mail: clebel@ucalgary.ca. Telephone: 403-955-7241; Fax: 403-955-2440

**Contact information of authors:**

Claire Donnici, BHSc; Email: claire.donnici@ucalgary.ca

Xiangyu Long, Ph.D.; Email: xiangyu.long@ucalgary.ca

Deborah Dewey, Ph.D.; Email: dmdewey@ucalgary.ca

Nicole Letourneau, Ph.D.; Email: nicole.letourneau@ucalgary.ca

Bennett Landman, Ph.D.; Email: bennett.landman@vanderbilt.edu

Yuankai Huo, Ph.D.; Email: [yuankai.huo@vanderbilt.edu](mailto:yuankai.huo@vanderbilt.edu)

**Keywords:** Passive-viewing functional magnetic resonance imaging, functional connectivity, amygdala, prenatal anxiety, neurodevelopment, child brain, APrON


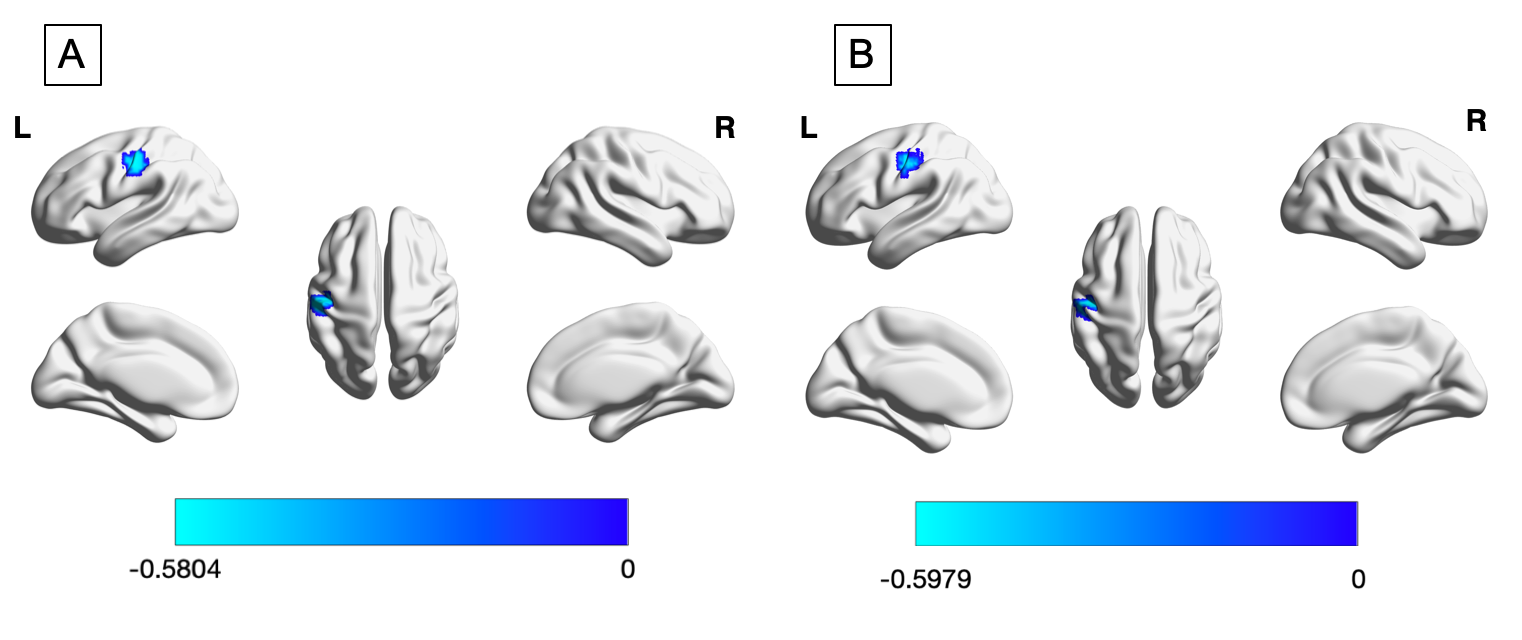


**Supplementary Figure 1. Relationship between maternal prenatal anxiety and amygdala functional connectivity at voxelwise p < 0.001**

At a voxelwise p-value of 0.001, maternal prenatal anxiety was significantly related to a cluster in the left postcentral gyrus and left inferior parietal lobule before controlling for postpartum depression and anxiety (n = 54; Cluster size: 233 voxels; rho = -0.58, p = 4.0 x 10^-6^; Peak: -49, -23, 36 – left inferior parietal lobule) (A). After controlling for maternal postpartum depression and anxiety, a slightly smaller version of this cluster was observed (n = 50; Cluster size: 190 voxels; rho = -0.56, p = 2.0 x 10^-5^; Peak: -46, -17, 39 – left postcentral gyrus) (B).
